# Lipid Transport and Metabolism in Healthy and Osteoarthritic Cartilage

**DOI:** 10.3390/ijms141020793

**Published:** 2013-10-16

**Authors:** Amanda Villalvilla, Rodolfo Gómez, Raquel Largo, Gabriel Herrero-Beaumont

**Affiliations:** 1 Osteoarticular Pathology Laboratory, IIS Fundación Jiménez Díaz, Madrid 28040, Spain; E-Mails: rlargo@fjd.es (R.L.); gherrero@fjd.es (G.H.-B.); 2 Musculoskeletal Research Group, Institute of Cellular Medicine, Newcastle University, Newcastle upon Tyne NE2 4HH, UK; E-Mail: rodolfo.gomez@newcastle.ac.uk

**Keywords:** chondrocyte, cartilage, osteoarthritis, lipid, cholesterol, nutrition

## Abstract

Cartilage is an avascular tissue and cartilage metabolism depends on molecule diffusion from synovial fluid and subchondral bone. Thus, nutrient availability is limited by matrix permeability according to the size and charge of the molecules. Matrix composition limits the access of molecules to chondrocytes, determining cell metabolism and cartilage maintenance. Lipids are important nutrients in chondrocyte metabolism and are available for these cells through *de novo* synthesis but also through diffusion from surrounding tissues. Cartilage status and osteoarthritis development depend on lipid availability. This paper reviews lipid transport and metabolism in cartilage. We also analyze signalling pathways directly mediated by lipids and those that involve mTOR pathways, both in normal and osteoarthritic cartilage.

## 1. Introduction

Lipid content in cartilage has been studied for many years. In the 1960s, Stockwell found that lipids in articular cartilage account for 1%, although this content was not modulated by age or sex [1]. While glucose is the main source of energy in chondrocytes [2], lipids in cartilage are necessary for cells as a source of energy but also to be incorporated as structural components and signalling molecules.

Cholesterol and fatty acids are the lipids that have been most frequently linked to cartilage physiopathology [3]. However, less is known about how these molecules can reach the chondrocytes and, once there, how lipids affect chondrocyte metabolism.

Proteins necessary for fatty acid metabolism and cholesterol biosynthesis, such as acetyl-coenzyme A acetyltransferase 1 (ACAT1), cytochrome P450 oxidase, family 51, sub-family A, polypeptide 1 (CYP51A1), 3-hydroxy-3-methylglutaryl-coenzyme A synthase 1 (HMGCS) or low density lipoprotein receptor (LDLR), have been detected in human chondrocytes [4]. In addition, lipids in joint fluid can also penetrate into cartilage [5]. Therefore, lipids may be available for chondrocytes directly from synovial fluid or by *de novo* synthesis.

Nevertheless, cartilage is an avascular tissue, so lipids need to travel through its compact matrix reaching the cells, which represent only about 2%–5% of total tissue, for supplying the chondrocyte metabolism. Therefore to better understand the role of lipids in chondrocyte metabolism it is necessary to analyze how these molecules are supplied to chondrocytes and afterwards to study lipid effects on cell metabolism.

## 2. Transporting Lipids through the Cartilage

Articular cartilage matrix is composed of 10%–30% collagen and 3%–10% proteoglycans and other minor glycoproteins and lipids. Proteoglycans, with a high anionic charge, provide the matrix with osmotic properties that allow cartilage resistance to loading, while also interacting with collagen to establish a network [6,7]. Therefore, cartilage matrix is basically a high negative charged network with large swelling pressure and tensile stress, where water and dissolved electrolytes filling the pores account for between 60% and 85% of total weight [7]. This complex structure determines the ability of articular cartilage to resist compressive loads, but also determines how molecules penetrate this matrix.

Small neutral solutes can diffuse freely from joint fluid into cartilage, while ionic and larger molecules show altered movement through the matrix. As described above, cartilage matrix is negatively charged which means that small anionic molecules are partially excluded, and the opposite occurs with small cationic solutes. Moreover, the ability to penetrate the cartilage matrix depends on the valency, which determines molecule affinity for the matrix [8].

Proteoglycans are heterogeneously distributed throughout the matrix [9], and variations in tissue fixed charge density may influence solute diffusion, mostly for larger molecules [8]. The main role of proteoglycans in molecule diffusion was corroborated by digesting human articular cartilage with several proteases, although this process did not affect the circulation of small solutes. However, although collagen removal did not alter the diffusive properties of any molecule, cathepsin D and trypsin digestions increased the diffusion of large molecules [10].

Lipids are usually found in blood as lipoproteins or bound to carrier plasma proteins in order to increase their solubility. This way, lipids become part of a large complex which restricts their transport through cartilage since size is a determining factor for molecule diffusion [11]. However, as Arkill *et al.* [11] suggested, this is not always the case, since lauric acid dissociates from albumin in cartilage surface and independently diffuses into the cartilage. In fact, this fatty acid was detected in the entire cartilage, accumulating in the tidemark [11].

Despite a different matrix composition, mature and immature cartilage show similar permeability, at least for uncharged solutes [12]. However, mature joints present a calcified cartilage in the bone-cartilage interface that does not exist in immature joints. This natural barrier could strongly interfere with the diffusion of lipids and other nutrients from calcified to non-calcified cartilage. Taking into account that diffusion of large molecules through cartilage matrix may be restricted, it is very important to understand what the possible nutrient sources in the joint are. Several studies have been carried out considering two alternatives: diffusion from synovial fluid and interchange from subchondral bone (SB).

There are big controversies about the SB as a source providing nutrients such as lipids to the cartilage, with the calcified cartilage in the bone-cartilage interface of mature joints being an important point in this debate. No nutrient diffusion from SB has been found in mature rabbits [13–15]; however, transport of molecules from SB has been shown in horses and mice [16,17]. In fact, mice present non-mineralized regions in calcified cartilage, forming pores to allow solute transport between cartilage and SB in mature joints [16].

Regarding human cartilage, it was firstly suggested that vascular channels penetrating from SB allowed molecule diffusion through calcified cartilage in human mature joints [18]. More recently the chondro–osseous junction in mature human cartilage was described as a complex 3D structure which presents a clearly defined tidemark that follows uncalcified cartilage prolongations crossing calcified cartilage to contact with bone and bone marrow vessels, allowing molecular trafficking [19].

Unlike subchondral bone, synovial fluid is widely accepted as a main source of molecules for articular cartilage metabolism [20,21]. In fact, Wang *et al.* [20] found that synovial fluid, but not SB, provides enough nutrients to maintain cartilage structure and function in mature rabbits.

In this regard, it is noteworthy that joints are subjected to compression and cyclic loading *in vivo*, modifying molecule diffusion from synovial fluid. Although molecule diffusion occurs in the absence of mobilization, fluid stirring and convection and cyclic loading are important for the diffusion of small solutes but even more for large solutes [22–24].

### 2.1. Osteoarthritis and Lipid Availability

Osteoarthritis (OA) is characterized by high levels of proteases that degrade cartilage matrix [25]. As stated above, this process involves increased cartilage permeability, which in turn could contribute to OA evolution [10]. Matrix components synthesized by chondrocytes in an attempt to repair the damaged cartilage could be lost in the joint space, being an unproductive effort [10]. Moreover, deleterious agents such as toxins and immunoglobulins, usually excluded from the cartilage, could reach chondrocytes and induce metabolic changes [10]. In fact, OA synovial fluid presents high levels of plasma proteins, which could easily reach chondrocytes and induce proinflammatory responses [26] (Table 1).

Moreover, OA cartilage does not only receive high levels of molecules from synovial fluid due to an increase in an aberrant permeability. Pan *et al.* found that during OA the cross-talk between SB and articular cartilage is enhanced due to an increase in the number of vessels invading the calcified cartilage [27]. In turn, immobilization and disability due to pain in OA patients may also affect cartilage permeability and therefore nutrient availability for the chondrocytes [22] (Table 1).

## 3. Lipids in Normal Cartilage

As stated above, human chondrocytes express several proteins for fatty acid metabolism and cholesterol biosynthesis. These molecules are up-regulated during chondrogenesis, indicating a high cholesterol biosynthesis in these cells [4]. In fact, cholesterol biosynthesis is required for expression of Ihh in rat chondrocytes and for normal growth plate chondrogenesis in rats [28,29].

On the other hand, leptin-like oxidized LDL (oxLDL) receptor 1 (LOX-1) [30] and Liver X receptor (LXR) [31] have been detected in human cartilage. These data indicate that chondrocytes are indeed able to modulate lipid homeostasis in the cartilage by sensing and transporting lipoproteins from the extracellular matrix.

Synovial fluid is an ultrafiltrate of plasma. However, plasma lipids are transported in large complexes so that human synovial fluid presents very low concentrations of lipoproteins in contrast to plasma [32,33]. It is important to note that the inflammation and vascular permeability of synovium determine the levels of lipoproteins in synovial fluid. Thereby, the ratio of synovial fluid to plasma of ApoA1, ApoB and Lp(a) is about 1.5 times higher in inflammatory arthritides like rheumatoid and psoriatic arthritis than in OA [33].

Despite being able to penetrate into cartilage, lipid content constitutes less than 1% of the total tissue weight [1]. High cholesterol content is found in chondrocyte cell membrane [34], indicating the importance of this lipid for structural maintenance in chondrocytes. In addition, the phytosterol stigmasterol is able to integrate in the chondrocyte membrane during culture [35]. On the other hand, palmitic, oleic and linoleic acids (16:0, 18:1n-9 and 18:2n-6) account for almost 85% of total fatty acid content in cartilage [36]. Moreover, their presence in cartilage is subjected to age-related changes. Studies both in humans and sheep demonstrated that fetal cartilage differs in lipid composition from mature cartilage. Indeed, human mature cartilage showed decreased monounsaturated and ω-3 fatty acids, while saturated fatty acids were increased [37]. In addition, arachidonic acid (20:4n-6) is higher in fetal cartilage meanwhile linoleic acid (18:2n-6) increases in mature cartilage (Table 2) [37]. Moreover, cultured growth plate chondrocytes present higher content in lipids than cultured articular chondrocytes [38].

It is noteworthy that, besides physiological modification of lipid content, the fatty acid composition in cartilage may be modulated by dietary lipids intake [39,40]. Diets containing high levels of ω-3 fatty acids lead to a decrease in arachidonic (20:4n-6) and linoleic acids (18:2n-6) and an increase in eicosapentaenoic acid (20:5n-3) in cartilage [39,40]. In addition, Nagao *et al.* demonstrated that extracellular fatty acids modulate intracellular lipid composition in cultured chondrocytes [41].

As stated above, cholesterol plays an important role as a structural molecule. However, the main effect of fatty acids in cartilage is through its conversion to eicosanoids. Both ω-3 and ω-6 fatty acids are substrates for cyclooxygenase and lipoxygenase enzymes, which synthesize prostaglandins and leukotrienes. These products derived from ω-3 fatty acids show anti-inflammatory properties, while those derived from ω-6 are pro-inflammatory and pro-thrombotic [42]; in fact, inhibition of cyclooxygenase results in less inflammatory mediators [43,44]. However, ω-6 fatty acids are also important for membrane structure and function, so it is necessary to maintain an appropriate ratio of ω-6 to ω-3, with 4:1 to 1:1 recommended [45].

Phospholipids are also important for normal cartilage maintenance. Besides being cell membrane constituents, phospholipids are key molecules in synovial fluid, playing a main role in joint lubrication to protect cartilage surfaces. In fact, phosphatidylethanolamine, phosphatidylcholine and sphingomyelin are major components of the articular cartilage boundary lubricant [46]. In addition to the presence of these phospholipids in the joint fluid, free fatty acids may be incorporated by chondrocytes into phosphatidylcholine (PC), phosphatidylethanolamine (PE), phosphatidylinositol (PI) and triacylglycerol (TG) [41,47].

Therefore, lipids are essential for cartilage physiology, and modifications in their availability and metabolism may have pathological consequences.

## 4. Lipids in Osteoathritic Cartilage

In recent years, OA has been linked to metabolic syndrome, which is characterized by dyslipidemia [48]. In fact, several studies have found an association between hypercholesterolemia and OA [49–51]. Therefore, some epidemiological studies have tried to elucidate the possible relationship between statin treatment to reduce serum cholesterol levels and OA development. However, the interpretation of the results of these studies presents some limitations regarding the evaluation of OA progression [52], leading to different conclusions. Clockaerts *et al.* [53] and Kadam *et al.* [54] found a reduced progression of knee OA in statin users; on the contrary, Riddle *et al.* [55] did not find an improvement in knee pain, function or structural progression, while Beattie *et al.* [56] concluded that statins did not influence the progression of an established OA.

Despite these controversies, the potential of statins to reduce OA has been observed *in vitro* and in animal models. In fact, statin treatment reduced OA progression in rats [57] and mice [58], and decreased pro-inflammatory and catabolic mediators in cultured chondrocytes [59–61]. However, cholesterol biosynthesis plays an important role in chondrogenesis [28,29]. Accordingly, these beneficial effects of statins have been related to reduced protein geranylgeranylation rather than inhibition of cholesterol synthesis itself [60,62].

Several studies have shown the beneficial effects of ω-3 fatty acids in inflammatory diseases such as rheumatoid arthritis (RA), recommending their use as part of the normal diet of patients [42]. However, only a few studies, without conclusive results, have been carried out in OA. Treating OA patients with cod liver oil, which contains high levels of eicosapentanoic acid (20:5n-3), in addition to non-steroidal anti-inflammatory drugs (NSAIDs) for 24 weeks showed no benefits *versus* olive oil [63]. However, the anti-inflammatory properties of several olive oil components could have attenuated the differences due to fatty acid composition [64]. In keeping with the lack of cod liver oil effect, Wang *et al.* found a correlation between ω-6 fatty acids intake and development of bone marrow lesions, but not with cartilage volume or damage [65]. In contrast, a recent study showed that total ω-3 fatty acids and the specific docosahexanoic acid (22:6n-3) were inversely correlated with patellofemoral cartilage loss, while ω-6 fatty acids showed no association [66]. Moreover, an animal model of spontaneous OA fed with a ω-3 fatty acids-enriched diet showed a lower pathology score, with increased glycosaminoglycan content, reduced denatured type II collagen, and reduced MMP-2 activity [67]. Very recently, Huang *et al.* found that decreasing ω-6 to ω-3 ratio by endogenous conversion of ω-6 to ω-3 fatty acids in mice delayed OA development [68]. Therefore, a beneficial effect of ω-3 fatty acids for OA cartilage may exist, although further *in vivo* and *in vitro* studies are needed to demonstrate it.

As stated above, phospholipids are important in joint lubrication. However, most of the phospholipids present in synovial fluid appear increased in OA, which could assist OA pathogenesis in modulating inflammatory responses [69]. In addition, high levels of phospholipase A2 are present in OA synovial fluid [70], where the main sources of this enzyme are the chondrocytes [71]. In fact, cytokines present in OA joints such as IL-1 are able to activate phospholipase A2 in chondrocytes, suggesting that this enzyme may play a role in the OA development [72]. Moreover, the activity of phospholipase A2 in OA synovial fluid could contribute to a release of fatty acids, which penetrate the cartilage matrix more easily than large complexes [11].

As with synovial fluid, lipid accumulation has been described in articular cartilage during OA development, despite the fact that diet association in OA still needs clarification. In fact, using imaging mass spectrometry, Cillero-Pastor *et al.* [34] have recently shown that cholesterol and fatty acids specifically accumulate in the superficial area of OA cartilage. Moreover, reduced expression of LXR and ApoA1 has been described in human OA cartilage, leading to impaired cholesterol efflux and finally intracellular lipid deposits in OA chondrocytes [31]. In fact, ApoA1^−/−^ mice spontaneously developed OA when fed a western-type diet due to alterations in HDL metabolism [73]. Changes in cartilage lipid composition are correlated with disease severity, with increases in total fatty acid of 440% in the advanced stages of OA [36]. Lippiello *et al.* suggested that high levels of arachidonic acid (20:4n-6) in OA could be related to the elevated eicosanoid synthesis usually found in this disease [36]. Therefore, altered lipid metabolism could be a risk factor and/or consequence of OA [34].

Lipid deposition in cartilage during OA has more consequences than merely accumulation itself. OA is characterized by an increase in reactive oxygen species, which are responsible for lipid peroxidation [74]; thus, lipid peroxidation products are usually found in joints from these patients [75,76]. Several studies have reported that these molecules contribute to cartilage degradation and OA pathogenesis, since they induce collagen oxidation and cleavage and MMP-13 activity [74,76]. Thereby, antioxidant treatment may be useful for OA not only by reducing reactive oxygen species [77] but also lipid peroxidation.

## 5. Lipids and Chondrocyte Metabolism

**Figure 1:**
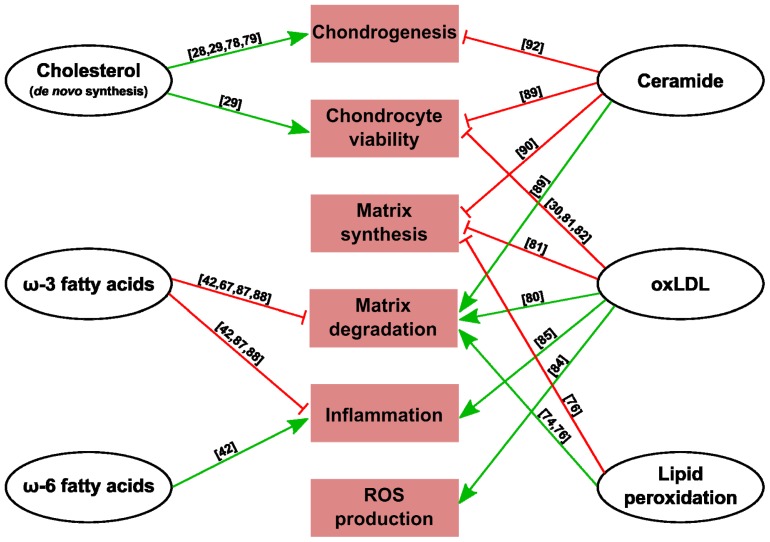
Summary of lipid actions in cartilage metabolism and response to stress. Green arrows indicate activation and red arrows inhibition of the processes shown in the squares. References are indicated in brackets.

Besides its structural role, cholesterol is also an important signal in chondrocyte biology. LXR is a sensor of oxygenated cholesterol derivatives, which activates transcription of important genes to protect cells from cholesterol overload. This receptor is modulated during chondrocyte differentiation and regulates cholesterol homeostasis during this process [78]. In fact, cholesterol signalling stimulates *in vitro* chondrocyte hypertrophy through nuclear receptor retinoid related orphan receptor-α (Ror-α) expression [79], and it is also necessary for apoptosis protection and Ihh expression during chondrogenesis and growth plate development [28,29] (Figure 1).

As stated above, OA chondrocytes express LOX-1, a scavenger receptor for oxLDL. oxLDL has been detected in human OA and RA cartilage [80,81], and stimulates senescence in human chondrocytes *in vitro* [82] by decreasing cell viability [30,81]. Moreover, this molecule induces reactive oxygen species production and hypertrophic-like changes in bovine chondrocytes [83,84], reduces proteoglycan synthesis in human chondrocytes [81] and increases MMP-3 production in human cartilage explants [80] and MCP-1 expression in human chondrocytes [85] (Figure 1).

The effects of fatty acids on chondrocyte metabolism support the potential benefits of adequate lipid content in diet. During *in vitro* experiments, bovine chondrocytes rapidly incorporated both arachidonic acid (20:4n-6) and oleic acid (18:1n-9). However, only arachidonic acid elicited metabolic changes, with increased matrix and prostaglandin synthesis [86]. Moreover, unlike arachidonic (20:4n-6), linoleic (18:2n-6), oleic (18;1n-9) and palmitic (16:0) acids, ω-3 fatty acids decrease proteinases involved in cartilage matrix degradation, COX-2, IL-1α, IL-1β and TNFα expression in chondrocytes [87,88] (Figure 1). However, rats fed 10% menhaden oil, which contains high levels of ω-3 fatty acids, showed a decrease in linoleic (18:2n-6) and arachidonic (20:4n-6) acids together with low proteoglycan synthesis and irregularities in cartilage [39]. This study points to the importance of an appropriate ω-6 to ω-3 ratio, where a high ratio may be as harmful as a very low one.

Ceramide is another lipid that is increased in the synovial fluid of OA patients [69]. Ceramide might play a mediatory role in cartilage loss during OA due to increased chondrocyte apoptosis and by inducing proteoglycan degradation [89]. High levels of endogenous ceramide have been demonstrated to disrupt cartilage matrix homeostasis, resulting in down-regulation of type II collagen in articular cartilage [90,91]. In fact, acid ceramidase, by degrading ceramide, alters sphingolipid metabolism and improves chondrogenesis [92] (Figure 1).

The mammalian target of rapamycin (mTOR) signalling pathway integrates both intracellular and extracellular signals and serves as a central regulator of cell metabolism, growth, proliferation and survival. Nutrients and energy status modulate mTOR signalling, leading to modifications in protein and lipid synthesis, lipid and glucose metabolism and autophagy [93]. Although mTOR is a critical regulator of lipid biosynthesis, its role as a lipid-sensing molecule has been studied to a lesser extent [94]. Palmitic acid (16:0) induces insulin resistance through activation of mTOR in skeletal muscle cells and hepatocytes [95,96], and a high-fat diet also induced insulin resistance in skeletal muscle in rats by mTOR activation [96]. In addition, arachidonic acid (20:4n-6) activates amino acid-independent mTOR signalling in breast cancer cells, inducing proliferation and angiogenesis [97]; however, ceramide induces autophagy by inhibiting mTOR signalling in several cell types [98]. mTOR has also been implied in macrophages’ intracellular cholesterol homeostasis, regulating the expression of important genes for cholesterol metabolism such as ABCA1, LOX-1 and LXR [99]. In addition, cholesterol membrane content is a regulator of mTOR signalling in endothelial cells, since alteration in cholesterol distribution in plasma membrane blunts mTOR pathway [100].

Therefore, mTOR is related to lipid-induced responses in different cell types. This pathway can induce insulin resistance and modulate cell proliferation and angiogenesis in response to specific fatty acids, and membrane cholesterol also seems to be necessary for mTOR signalling. However, little is known about this signalling pathway in chondrocytes. It has been implicated in the process of chondrocyte differentiation, through modulation of autophagy and Ihh expression [101,102]. In fact, inhibition of mTOR by rapamycin prevents chondrocyte differentiation, showing an important role for mTOR in this process [79]. Autophagy has been proposed as a protective mechanism in normal cartilage; this way, aging and OA-related autophagy loss could be involved in cartilage damage during these situations [103]. Accordingly, autophagy induction by mTOR blockade using rapamycin in OA mice reduced disease severity, as shown by less cartilage degradation [104]. In contrast, Sasaki *et al.* found increased autophagy in OA chondrocytes, however, they also suggested that rapamycin-induced autophagy is a protective mechanism from stresses [105]. According to results in other cell types, arachidonic acid (20:4n-6) has been described as an activator of mTOR signalling in mice chondrocytes, while docosahexaenoic acid (22:6n-3) reverts this effect [68]. This recent study further confirms the beneficial role of ω-3 fatty acids in chondrocytes, inhibiting mTOR and activating autophagy as a protective mechanism for cartilage maintenance [68].

## 6. Conclusions

Lipids such as phospholipids, cholesterol and fatty acids are present in articular cartilage and synovial fluid. Chondrocytes can synthesize these molecules, however, dietary lipids may also reach the cartilage, modify its composition and be incorporated in chondrocyte metabolism and structures. In fact, impaired cholesterol efflux and intracellular lipid deposits, as well as fatty acid accumulation in cartilage, have been related to OA development.

Several studies have suggested that anti-inflammatory and anti-catabolic properties of ω-3 fatty acids, as well as their capacity to inhibit mTOR signalling and promote autophagy, may be used to treat OA symptoms and progression. Therefore, an appropriate lipid intake could contribute to improving lipid balance in cartilage, preventing chondrocyte metabolism alterations and cartilage damage.

## Figures and Tables

**Table 1:** Altered cartilage permeability during osteoarthritis (OA).

Altered cartilage permeability	Events related to OA	References
**Causes**	Increased protease activity	[25]
	Increased subchondral vessels invading calcified cartilage	[27]
	Joint immobilization	[22]

**Consequences**	Loss of matrix components in the joint space	[10]
	Access of deleterious agents (toxins, immunoglobulins)	[10]
	Access of proinflammatory plasma proteins	[26]

**Table 2:** Fatty acid composition in normal cartilage.

Cartilage maturation stage	Cartilage composition	References
**Mature cartilage**	↑ saturated fatty acids	[37]
	↑ linoleic acid	[37]

**Fetal cartilage**	↑ ω-3 fatty acids	[37]
	↑ mono-unsaturated fatty acids	[37]
	↑ arachidonic acid	[37]
